# EEMD Independent Extraction for Mixing Features of Rotating Machinery Reconstructed in Phase Space

**DOI:** 10.3390/s150408550

**Published:** 2015-04-13

**Authors:** Zaichao Ma, Guangrui Wen, Cheng Jiang

**Affiliations:** 1 School of Mechanical Engineering, Xi’an Jiaotong University, No.28 Xianning West Road, Xi’an 710049, China; E-Mails: mazaichao@163.com (Z.M.); jiangcheng422@163.com (C.J.); 2 State Key Lab for Strength and Vibration of Mechanical Structures, Xi’an Jiaotong University, Xi’an 710049, China; 3 School of Mechanical Engineering, Xinjiang University, No.1043 Yanan Road, Wulumuqi 830047, China

**Keywords:** PSEEMD, EEMD, mode mixing, amplitude of added noise, weak features

## Abstract

Empirical Mode Decomposition (EMD), due to its adaptive decomposition property for the non-linear and non-stationary signals, has been widely used in vibration analyses for rotating machinery. However, EMD suffers from mode mixing, which is difficult to extract features independently. Although the improved EMD, well known as the ensemble EMD (EEMD), has been proposed, mode mixing is alleviated only to a certain degree. Moreover, EEMD needs to determine the amplitude of added noise. In this paper, we propose Phase Space Ensemble Empirical Mode Decomposition (PSEEMD) integrating Phase Space Reconstruction (PSR) and Manifold Learning (ML) for modifying EEMD. We also provide the principle and detailed procedure of PSEEMD, and the analyses on a simulation signal and an actual vibration signal derived from a rubbing rotor are performed. The results show that PSEEMD is more efficient and convenient than EEMD in extracting the mixing features from the investigated signal and in optimizing the amplitude of the necessary added noise. Additionally PSEEMD can extract the weak features interfered with a certain amount of noise.

## 1. Introduction

Signal processing techniques, which discover the essential rules contained in a mechanical dynamic system, are widely used to realize the vibration analysis, the fault identification, and the status estimation of systems [1,2,3,4,5,6,7]. A series of methods based on inner product transformation [8,9] exhibit effective usage of *a priori* knowledge for the non-linear and non-stationary phenomenon encountered typically in practice. The inner product transformation can be adopted to obtain features of the mechanical system by measuring the correlation between vibration signal and a pre-defined kernel function. However, a description of continuous and instantaneous variation of a dynamic system with non-linear and non-stationary properties may be generally absent due to the lack of adaptivity. On the other hand, the low intelligence and flexibility attributed to the non-adaptivity may lead to the requirement of a large amount of *a* *priori* knowledge or new kernels for a suitable match of the inner product. 

Empirical Mode Decomposition (EMD) [10], proposed by Huang, enables adaptive feature extraction from signals and is applied in the vibration analyses for rotating machinery [11,12,13,14,15]. EMD is able to decompose a complex signal into a series of Intrinsic Mode Functions (IMFs) whose frequencies arrange from high to low amplitude and IMFs are characterized by completeness and low redundancy. This method measures the oscillation of signals with the instantaneous frequency and thus establishes a new time-frequency framework with IMFs. Basis function expressed by IMF is determined with the local and global information from the signal itself, thus avoiding the pre-defined kernels derived from the inner product transformation. Nevertheless, mode mixing, which is defined as either a single IMF consisting of components with wide disparate scales or a component with a similar even identical scale residing in different IMFs, will be generated from anomalous incidents, such as the noise interference and intermittent composition [16,17,18,19]. Consequently, Wu introduced white noise to assist EMD named ensemble empirical mode decomposition (EEMD) [18], which relieves mode mixing efficiently. This new method has been employed in vibration analysis of rotating machinery frequently for the more advance independent mode extraction than EMD [19,20,21]. However, in EEMD, the elimination degree of mode mixing has to be determined by the amplitude of added noise. Although some qualitative and quantitative strategies for mode mixing have been proposed [17,18,21], little research has been done on the independent extraction of components. In this paper, we develop Phase Space Ensemble Empirical Mode Decomposition (PSEEMD) to improve EEMD used in the vibration analyses for rotating machinery. By our PSEEMD, the independent feature extraction can be realized, which meets the requirement of practical applications.

It is obvious that the extraction results from non-linear and non-stationary signals, combined with noise, approach linear and stationary information. Consequently, the nature of a dynamic system may be reconstructed with these brief signal components approaching linear and stationary information through an appropriate expression, Phase Space Reconstruction (PSR) [22,23]. Then, it is possible to realize the independent feature extraction by investigating the topological property of a manifold presenting in a phase space.

Studies on PSR were conducted as early as 1980 [22,23]. Since then, PSR has been developed gradually with the coordinate delay method for expressing a data manifold in a phase space [24]. In this framework, the embedding dimension and the time delay are two critical parameters, which can be optimized by several conventional methods [25,26,27,28,29,30,31]. In this paper, we note that PSR realized by Hankel matrix could represent the topological structure of IMFs competently. Moreover, the mixing features can be extracted independently and the optimization of added noise can be performed with the linear manifold learning (ML) method [32]. Our research indicates that the use of the ML method will be beneficial to the weak feature extraction. On the basis of our findings, we propose a new adaptive EEMD model named PSEEMD. In this model, IMFs selected are extended to attractors first and then independent features are extracted from the attractors, and modified IMFs will be reconstructed with the new features afterwards, thus leading to the parameter optimization and higher capability of weak feature extraction in the vibration analyses for rotating machinery.

The rest of this paper is organized as follows. In Section 2 we briefly describe the EEMD and the related concept. The original contribution of the paper is presented in Section 3, where we give a detailed description of PSEEMD and a noiseless simulation signal with multi-component is utilized to validate the feasibility of PSEEMD. In Section 4 we briefly illustrate the general information of a rotor test bench and detailed installation of rubbing. Then, PSEEMD is supplied with how to deal with mode mixing through a rubbing signal with strong noise interference. Conclusions and possible extensions appear in Section 5.

## 2. Basic Conception

### 2.1. Ensemble Empirical Mode Decomposition (EEMD)

As a pioneer of the adaptive data analysis method, EMD was proposed by Huang [10]. This well-known method has been widely used for the feature extraction of non-linear and non-stationary signals in many fields, especially for vibration analysis of rotating machinery [33,34,35]. EMD, however, is subjected to mode mixing, which is defined as either a single IMF consisting of components with wide disparate scales, or a component with a similar even identical scale residing in different IMFs. On the basis of EMD, Wu and Huang developed EEMD to improve the independent extraction capability of signal components. As a noise-assisted data analysis method, EEMD defines the true IMF component as the mean of an ensemble of trials, and each trial contains the result of the signal plus a white noise of the finite amplitude decomposed by EMD [18]. The realization of EEMD is dominantly based on the idea that different white noise with the same statistical characteristics can be reduced or even cancelled out in the ensemble mean of enough trials. Consequently, the added white noise would populate the whole time-frequency space uniformly, taking advantage of the dyadic filter bank behavior of the EMD. The principle of EEMD can be seen in previous literature [18,21,36,37]. It is critical to guarantee that there are only persistent components when more trials are introduced into the ensemble. 

**Figure 1:**
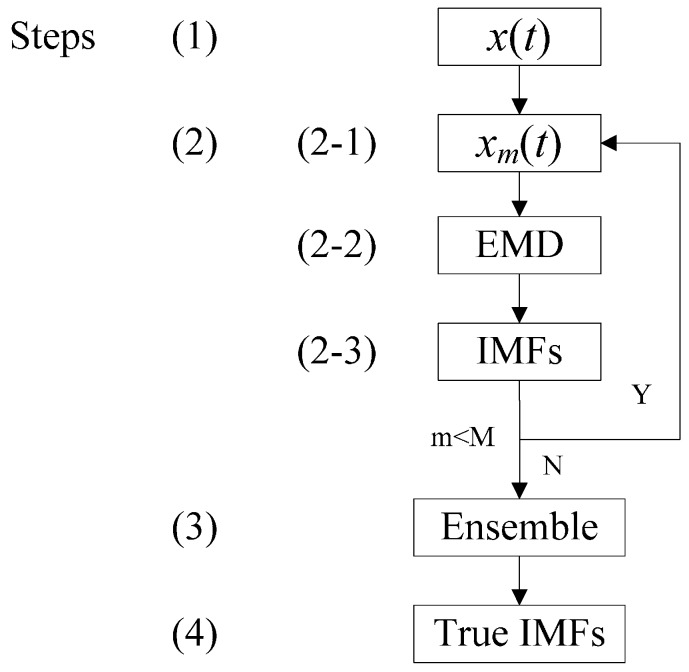
Flow of ensemble empirical mode decomposition (EEMD).

In order to satisfy the needs of comparison in the subsequent improvement, the whole flow of the EEMD algorithm and the description [18,21] are shown in Figure 1.

Steps:

(1) Initialize the ensemble number *M*, the statistical property of added white noise, and *m* = 1.
(2) Perform trails *M* times in a cycle.
  (2-1) Add the *m*th white noise with the given statistical property to the signal,
    (1) $$x_{m}(t)=x(t)+n_{m}(t)$$
    where *n_m_*(*t*) represents the *m*th added white noise, and *x_m_*(*t*) represents the noise-added signal of the *m*th trial.
  (2-2) Decompose the noise-added signal *x_m_*(t) into IMFs *c_i_*_,*m*_ (*i* = 1,2,…,*I*) and a residual series *r_m_* by EMD:
    (2) $$x_{m}\left(t\right)=\sum_{i=1}^{I}c_{i,m}+r_{m},i=1,2,...,I$$
    where *c_i_*_,*m*_ denotes the *i*th IMF of the *m*th trial, *I* is the number of IMFs, and *r_m_* represents the only residual volume.
  (2-3) If *m* < *M*, the flow performs back to (2-1), accompanying *m* = *m* + 1. Then, perform circulation from (2-1) to (2-3) until *m* = *M*.
(3) Calculate the ensemble mean *c_i_* of the *M* trials for each IMF and the residual *r* using the following equation.
  (3) $$\begin{array}{ll} \begin{array}{c} c_{i}=\frac{1}{M}\sum_{m=1}^{M}c_{i,m}\\ r=\frac{1}{M}\sum_{m=1}^{M}r_{m} \end{array} & ,m=1,2,...,M \end{array}$$
(4) Output all of the true IMFs which should satisfy the formula:
  (4) $$x\left(t\right)=\sum_{i=1}^{I}c_{i}+r,i=1,2,...,I$$

According to the flow chart and the description above, we can understand that mode mixing can be restrained by adding white noise to the investigated signal to change the distribution of extremes. In this process, the critical portion of the amplitude of added noise determines the restraining level. This parameter mainly follows the statistical regularity as follows [18]:

(5) $$\varepsilon_{n}=\frac{\varepsilon }{\sqrt{M}}\text{ or }\ln\varepsilon_{n}+\frac{\varepsilon }{2}\ln M=0$$

where *ε_n_* is the deviation between the original signal and the reconstruction result, *ε* represents the amplitude of added noise that we need to find out, and *M* is the ensemble number.

It can be seen from the formula that the precision of signal decomposition is proportional to the amplitude of added noise and inversely proportional to the ensemble number. Under ideal condition, the smallest *ε* and the largest *M* can ensurethe smallest *ε_n_*. However, in fact, the smallest *ε* will lead totoo small change of extreme distribution and the largest *M* will cause the higher complexity of the algorithm. To solve the above problem in practice, *ε* can be designed as 0.2 times of the standard deviation of the investigated signal generally according to noise added experiments. On the other hand, Wu, *et al*., also presented some empirical rules for complement: if the high frequency components are dominant in the investigated signal, *ε* can be slightly smaller while the low frequency components are dominant, *ε* can be slightly greater. Several researchers have conducted the investigation into the relationship between the high or low frequency components and *ε* [17,21].

Although much work has been done on the amplitude of added noise and some novel strategies have been adopted for developing the new ideas on the solution of parameter optimization, little information is available on the effect of mode mixing in parameter optimization. Here we propose an algorithm on the basis of PSR and ML.

### 2.2. PSR Based on Hankel Matrix

The theory of PSR [22,23] indicates that an attractor can be reconstructed by univariate time series. This model can state the dynamic properties of a complex system efficiently and offer a feasible way to understand and analyze a system. The embedding dimension and the time delay are two critical parameters that can be optimized by several conventional methods. In order to display the ideas in this paper, a simple expression of the Hankel matrix is adopted for lowering model complexity and performing signal reconstruction linearly by a high resolution [38] and the zero phase-shift.

Phase Space Reconstruction with the Hankel matrix has the form.

(6) $$X=\left[\begin{array}{cccc} X_{\text{1}} & X_{\text{2}} & \cdots & X_{l}\\ X_{\text{2}} & X_{3} & \cdots & X_{l+1}\\ \vdots & \vdots & & \vdots \\ X_{m} & X_{m+1} & \cdots & X_{m+l-1} \end{array}\right]$$

where l≥2, m≥2, m+l−1=N and *N* denotes the length of the univariate time series.

The vectors correspond to the points of the phase space with *m* dimensions. These points can be connected in order to obtain an attractor which can be expressed as an *m* × *l* matrix in Equation (6).

Singular Value Decomposition (SVD) is a conventional method to analyze the Hankel matrix. Note that SVD, however, is equivalent to a portion of Principle Component Analysis (PCA) [39]. To reduce the computational complexity and enhance the effectiveness, PCA can be employed for reducing the dimensions of the Hankel matrix and extracting the main features.

### 2.3. Manifold Learning Based on Principle Component Analysis (PCA)

Seung *et al*., indicated that the high order correlation generally exists among features of complicated modes in ML [32]. As a result, the low order statistical property of a data set presents obviously non-linear. This character can be expressed by a set of implicit variables whose dimensions are far below the sample dimensions. ML enables the linear statistical pattern recognition to be transformed to the non-linear one. PCA is one of the most conventional feature extraction methods in statistical pattern recognition [40,41] and also included in ML. This method transforms a group of variables ***X*** in *n* dimensional vector space into a new group of ***Y*** that the variance achieves maximum by an orthogonal matrix ***A*** to be found out. The maximum variance can be represented as an optimization problem of minimizing the mean square error (MSE) as follows.

(7) $$\min E\left({\left\|X-\sum_{i=1}^{m}Y\alpha_{i}\right\|}^{2}\right)$$

where *E*[·] represents the mathematical expectation, *α* is the projection direction included in ***A***.

Therefore, taking one of the new variables *Y_i_* as an example, its variance can be written as

(8) $$\begin{array}{l} Var(Y_{i})=E[Y_{i}^{2}]-E{\left[Y_{i}\right]}^{2}\\ =E[\alpha_{i}^{T}XX^{T}\alpha_{i}]-E{\left[\alpha_{i}^{T}X\right]}^{2}\\ =\alpha_{i}^{T}E[XX^{T}]\alpha_{i}-{\left(\alpha_{i}^{T}E[X]\right)}^{\text{2}}\\ =\alpha_{i}^{T}C\alpha_{i}-{\left(\alpha_{i}^{T}0\right)}^{2}\\ =\alpha_{i}^{T}C\alpha_{i} \end{array}$$

where ***C*** is the covariance matrix of ***X***.

Thus, the extreme problem under the condition of *α^T^α* = 1 can be converted to an unconstrained extreme problem as

(9) $$f(\alpha_{i})=\alpha_{i}^{T}C\alpha_{i}-v\alpha_{i}^{T}\alpha_{i}$$

where *v* is the Lagrangian multiplier.

Calculating the differential of Equation (9) and setting the result to be zero, the MSE can be deduced as a solution to the characteristic Equation (10).

(10) Cα−vα=0

The main features can be selected to be the original signal and then the goal of feature extraction can be achieved accordingly. 

Note that PCA is able to verify the feasibility of extracting the orthogonal features of the investigated signals. However, other embedding and manifold learning methods would be adopted instead of PCA if the investigated signal with certain non-linear and non-stationary property were difficult to analyze.

## 3. Independent Extraction for Mixing Features Based on PSEEMD

### 3.1. Preliminary Extraction of Signal Mode Based on EEMD

On the basis of the noise decoupling, EEMD is expected to decompose the signal into a set of IMFs with frequencies arranged from high frequency to low one. In vibration analyses of rotating machinery, IMFs with too high frequency would be the stochastic noise usually, and IMFs with too low frequency would be the trends, the false components, and the residual components. IMFs that represent the vibration property of rotating machinery can be obtained by eliminating the useless components.

The vibration signals of rotating machinery are mainly concentrated in the low frequency region. We remark that, according to the instruction of parameter criteria in Section 2.1 and our experiment data, the amplitude of added noise *ε* will be a proper value that is greater than 0.2 times of the standard deviation of the investigated signals, and the ensemble number *M* will be less than 100.

### 3.2. IMFs Reconstructed in Multi-Phase-Space

Different PSR methods are suitable for the different purposes. For the linear and stationary IMFs, we adopt the Hankel matrix mentioned in Section 2.2 to reconstruct the phase space in order to reduce the model complexity and reconstruct the signals linearly by a high resolution and the zero phase-shift. Obviously, a series of phase space named multi-phase-space (MPS) constructed by the multi-Hankel-matrix will be established with the selected IMFs, and the single components will offer a good chance to be extracted from these spaces which are approximately linear and stationary by post processing described in the next section.

### 3.3. Modified IMFs Established by Element Storage

Through the extended expression of IMFs in the multi-phase-space, manifold learning methods may be used to extract features of the investigated signal further. Non-linear manifold learning methods proposed recently seem to be fussy and inappropriate for feature extraction in multi-phase space with approximately linear and stationary characteristics. Consequently, the multi-manifold-learning (MML) with PCA, instead of complex and conventional SVD, is adopted here, tentatively, for the single elements extraction in multi-phase-space. A new set of “element storage” would be constructed with these single elements. By inspecting the relationship of these elements between spaces properly and combining the same kind of elements, the modified intrinsic mode function (MIMF) would be reconstructed. Thus the independent extraction of signal components would be realized accordingly. The overview of PSEEMD and its details are given in Section 3.4.

### 3.4. Overview of PSEEMD

**Figure 2:**
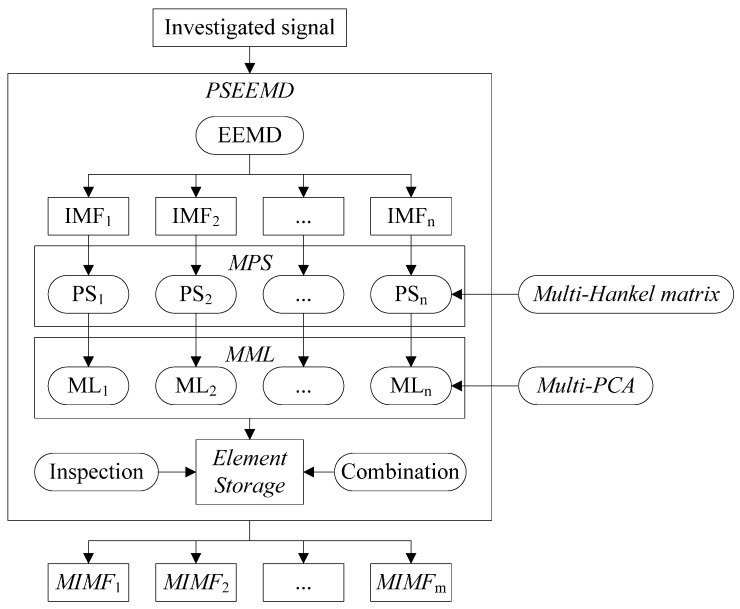
Flow of Phase Space Ensemble Empirical Mode Decomposition (PSEEMD).

Implementation procedure of PSEEMD is described as follows and the detailed computation flow is shown in Figure 2.

Step 1: Implement EEMD with the investigated signal and obtain the preliminary IMFs.
Step 2: Select required IMFs and construct MPS by the multi-Hankel-matrix.
Step 3: Extract elements from MPS by MML of Multi-PCA and develop the element storage.
Step 4: Inspect the element storage and combine the same kind of elements.
Step 5: Reconstruct MIMFs.

Notes:

No.1: The amplitude of added noise in EEMD is set to be 0.2 times of the investigated signal standard deviation or an optimized value. The following verification with PSEEMD in Section 3.5 indicates that almost no difference exists between these two arrangements.
No.2: Required IMFs should be selected according to some prior knowledge or indicators of the investigated signal.
No.3: The element storage may be enormous and some prior knowledge may be also used to inspect and combine the same kind of elements.

### 3.5. Simulation Verification

**Figure 3:**
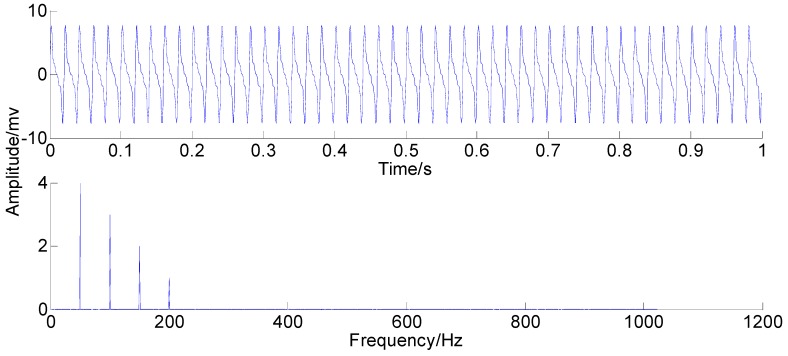
The simulation signal.

A simulation signal is adopted to explain the proposed PSEEMD in this section. Since rubbing is a typical fault in rotating machinery, the simulation signal mainly includes the fundamental frequency and harmonics of rubbing. Rubbing is defined as the impact when the interval between static part and spindle is tiny. This fault signal is commonly characterized with evident fundamental frequency, along with the integer harmonics and the fractional harmonics. The fundamental frequency accompanying integer frequency less than or equal to the quadruplicated frequency is a dominant feature presented by the running status of our present rotor test bench. A simulation signal constructed by the four frequency components is shown in Figure 3, which shows clearly the specified four frequency components in the frequency domain.

**Figure 4:**
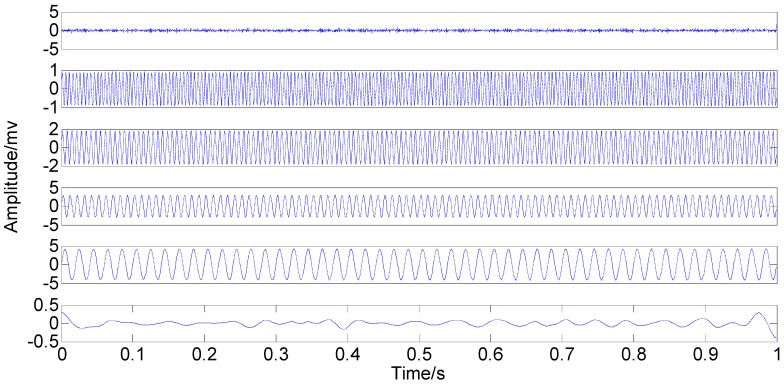
Modified Intrinsic Mode Functions (IMFs) in the time-domain.

**Figure 5:**
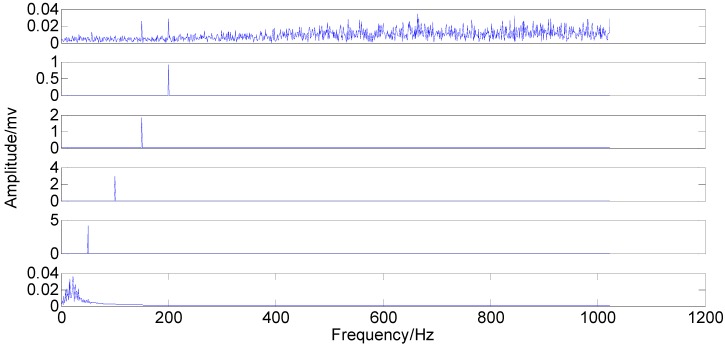
Modified IMFs in the frequency-domain.

With the original amplitude of added noise equal to 0.77, the ensemble number of 100 and the sifting number of 20, the PSEEMD method is employed to analyze the simulation signal and the decomposed first six Modified IMFs, both in the time-domain and the frequency-domain, are illustrated in Figure 4 and Figure 5, respectively.

**Figure 6:**
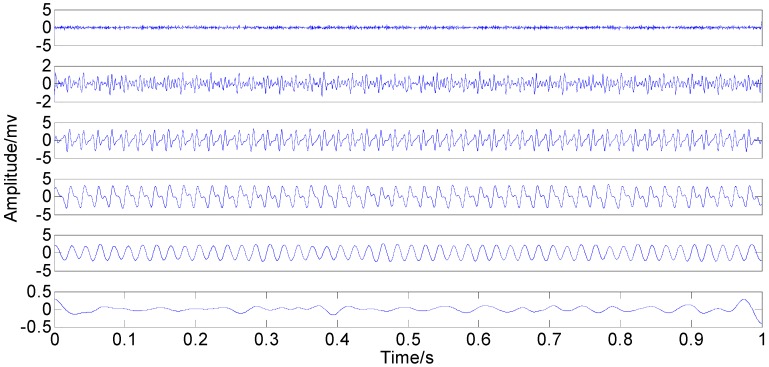
Original IMFs in the time-domain.

**Figure 7:**
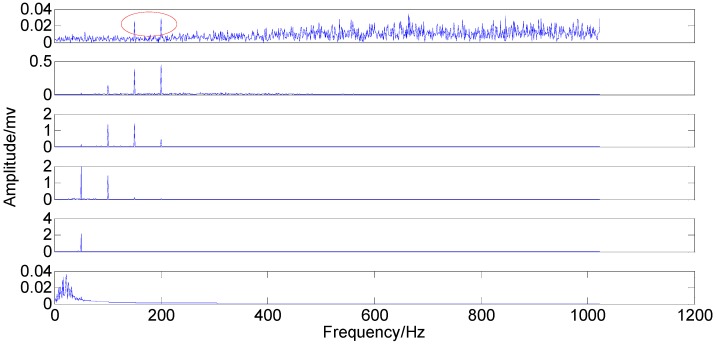
Original IMFs in the frequency-domain.

**Figure 8:**
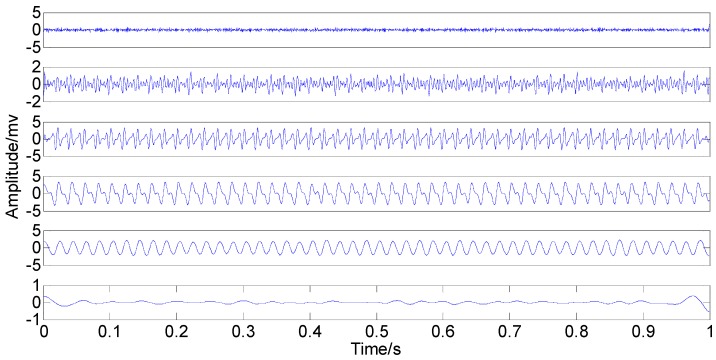
Optimized IMFs in the time-domain.

**Figure 9:**
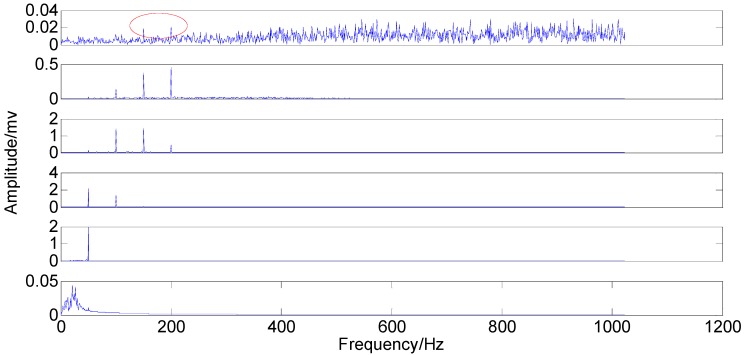
Optimized IMFs in the frequency-domain.

It can be seen from the two figures that MIMFs 1–6 correspond to the passband noise, the quadruplicated vibration frequency (1X), the triple vibration frequency (2X), the double vibration frequency (3X), the fundamental vibration frequency (4X), and the low frequency noise, respectively. It can also be observed from Figure 3 that MIMFs 2–5 almost correspond to the four components, respectively, showing that the four main components existing in the simulation signal are extracted independently by PSEEMD. For further comparison, the simulation signal is also analyzed by the original EEMD and the optimized EEMD, respectively. Here, the optimized EEMD is referred to as iterative search for an optimal *ε* in a proper interval whose lowerbound is greater than 0.2 times of the standard deviation of investigated signal. These two kinds of decompositions are shown in Figure 6, Figure 7, Figure 8 and Figure 9.

The comparison between the original IMFs in Figure 6 and Figure 7 and Optimized IMFs in Figure 8 and Figure 9 show that mode mixings are both serious and there is almost no difference between them, especially for the main components of IMFs 2–5. The original amplitude of 1X is approximately equal to 0.0256 and that of 2X is nearly 0.0284, while the optimized IMF1 amplitude of 1X decreases from the initial value to 0.01929 and that of 2X also declines to 0.02055—See the marked red circle in Figure 7 and Figure 9. This obvious change may be attributed to that the order of magnitude of IMF1 is much less than those of IMFs 2–5. Actually, components for other IMFs also increase or decrease slightly. This phenomenon suggests that some weak information transfers from the first IMF to the others. Therefore, EEMD with optimized *ε* only enables some weak information transfer rather than eliminating mode mixing. Moreover, the information transfer may be more obvious if the complexity of investigated signal increases. Thus, these results imply that neither the original EEMD nor the optimized EEMD can accurately extract independent information of the simulation signal.

On the basis of the results of the simulation and comparison, it appears that PSEEMD is able to obtain more accurate and independent IMFs than the original EEMD and the optimized EEMD.

## 4. Experimental Presentation for Feature Extraction of Faulted Rotor System

### 4.1. Brief Introduction of Experiment on Rotor System

The rotating machinery is a kind of critical equipment in the fields of electrical power, petrochemical complex, metallurgical industry and aerospace engineering. Contemporary rotating machinery is confronted with severe production conditions, complex and diverse equipment structure, and quick updating of physical and virtual function, which has raised a higher reliability requirement for the versatile and critical units of rotor systems. For the security and stability of the rotating machinery, it is expected to acquire breakthrough on the feature extraction and the identification of the running status, especially on the weak feature extraction for the rotor vibration signal with non-linear and non-stationary properties under the strong noise condition.

**Figure 10:**
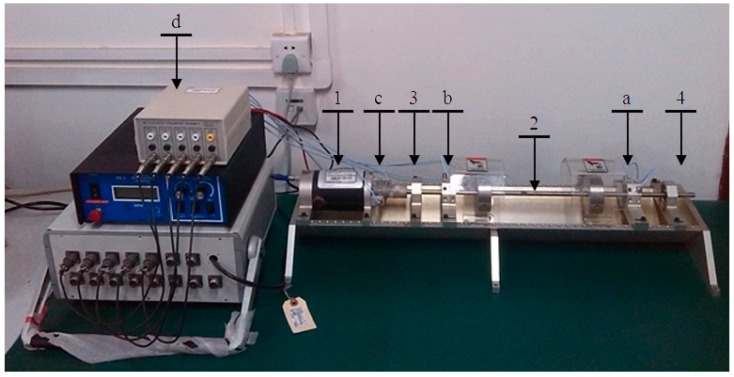
Bently RK4 rotor test bench. 1. motor, 2. rotor, 3. bearing, 4. foundation; a. sensors 1~2; b. sensors 3~4; c. sensors 5~6; d. data acquisition instrument.

Consequently, a Bently RK4 rotor test bench is adopted in our research. The main framework is given in Figure 10. This platform mainly consists of a rotor system and a vibration testing system. The rotor system includes a rotor, a motor, a pair of bearing and a foundation. The vibration testing system includes six groups of eddy current sensors and a set of data acquisition instrument connecting computer. Sensors 1–4 are divided into two groups for capturing the vibration signals located in the cross section with the directions of 45° and 135°. Sensor 5 is used to measure phase and sensor 6 is applied to capture the rotating speed. 

**Figure 11:**
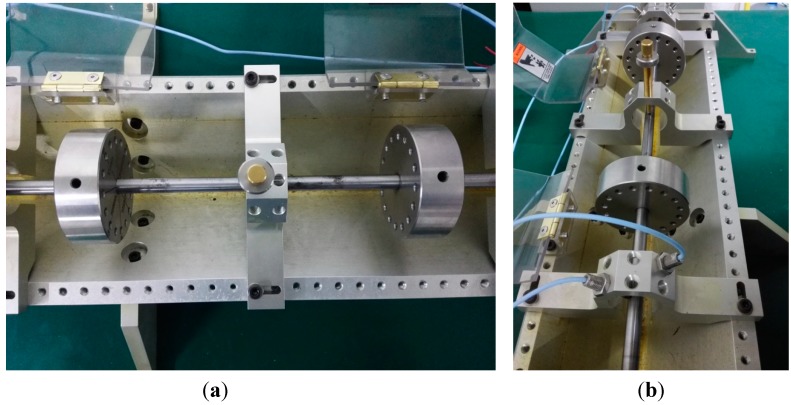
Installation effect of rubbing (a) vertical view; (b) side view.

A typical fault of rubbing is designed in our research to verify the feasibility of PSEEMD. Rubbing is defined as the impact when the interval between the static part and spindle is tiny, and this fault can be yielded by a supported design. The installation of the rubbing support is shown in Figure 11. The property of this rotor fault can be regarded as an extension of the simulation signal with an increased in non-linear and non-stationary features. The sampling rate is set to be 2048 Hz, the sampling number is set as 2048 points, and the rotating speed is set as 3000 rpm for safety.

### 4.2. Rubbing Features Extraction with PSEEMD

**Figure 12:**
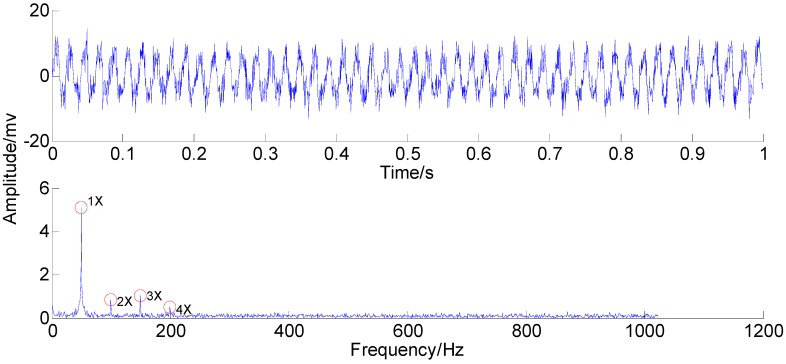
The rubbing signal in the time-domain and the frequency-domain.

The rubbing rotor vibration signal in the time-domain and the frequency-domain are shown in Figure 12.

**Figure 13:**
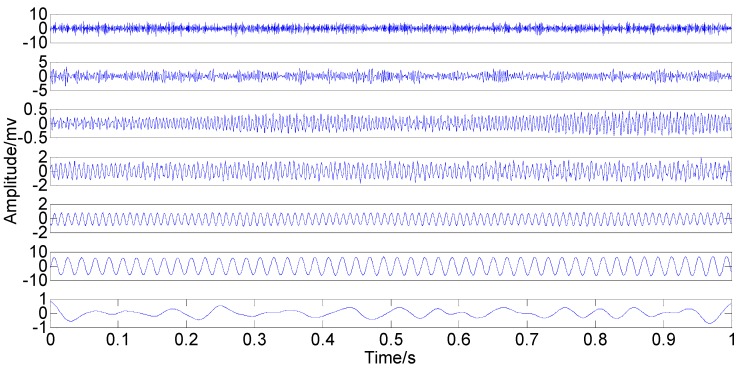
Modified IMFs in the time-domain.

**Figure 14:**
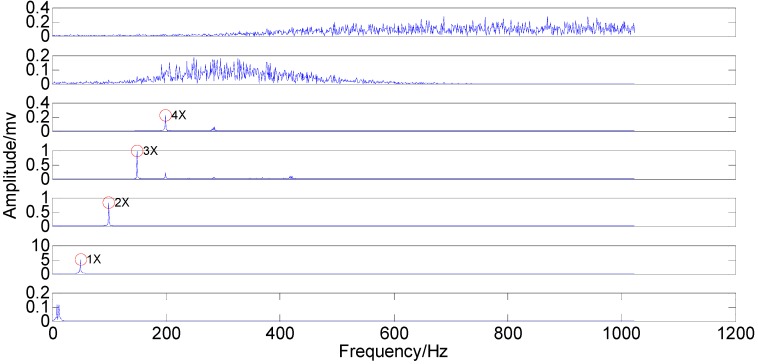
Modified IMFs in the frequency-domain.

According to the waveform of the rubbing signal in the time-domain, it is difficult to find detailed information of features due to the interference of the strong noise, except the global oscillatory amplitude. Although the primary component distribution can be seen in the frequency-domain, 2X, 3X and 4X are exactly the weak features, especially 4X. This information indicates non-linear and non-stationary complexity of the rubbing features. In order to solve this feature extraction problem and manifest the feasibility of PSEEMD, decomposition of the rubbing signal by PSEEMD has been computed, as shown in Figure 13 and Figure 14. The amplitude of added noise is equal to 1.09, the ensemble number is 100 and the sifting number is 20.

From the two figures, it can be noted that MIMF1 and MIMF2 represent high frequency noises lying in the first two regions. MIMF3, MIMF4, MIMF5 and MIMF6 are corresponding to 4X, 3X, 2X and 1X, respectively, when MIMF6 and MIMF5 exhibit great smoothness of sinusoidal wave. Furthermore, MIMF4 and MIMF3 show inferior smoothness but still represent primary features. This distinction is due to the noise strength. Taking MIMF6 and MIMF3 as an example, the 1X of MIMF6 is from those of IMF5 and IMF4, which are far away from the strong noise region. Nevertheless, the 4X of MIMF3 is directly from that of IMF2, which is characterized with strong noise. Note that MIMFs 3–6 are almost equal to the four marked components in Figure 12. Like the signal simulation results, primary components existing in the investigated signal are extracted independently. As a result, PSEEMD can still extract the primary and the weak information independently from the strong noise, although the noise disturbs the feature extraction.

**Figure 15:**
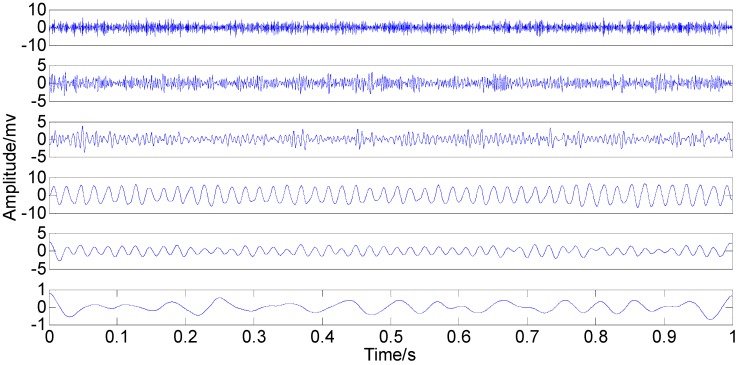
Original IMFs in time-domain.

**Figure 16:**
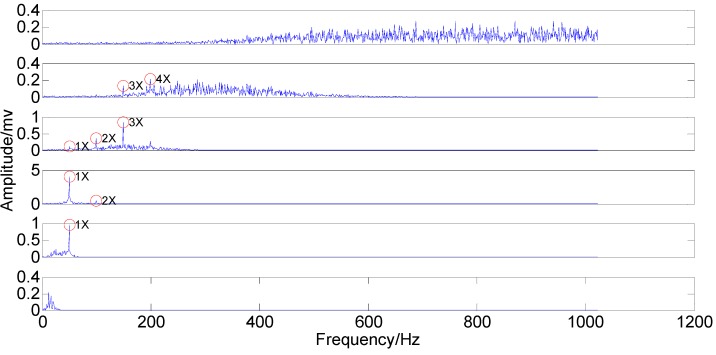
Original IMFs in the frequency-domain.

**Figure 17:**
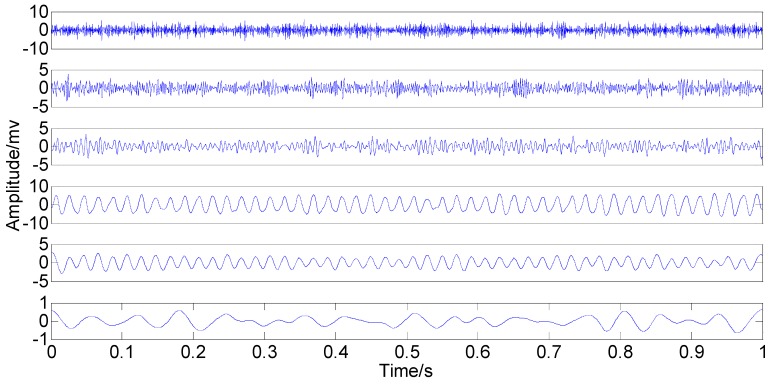
Optimized IMFs in the time-domain.

**Figure 18:**
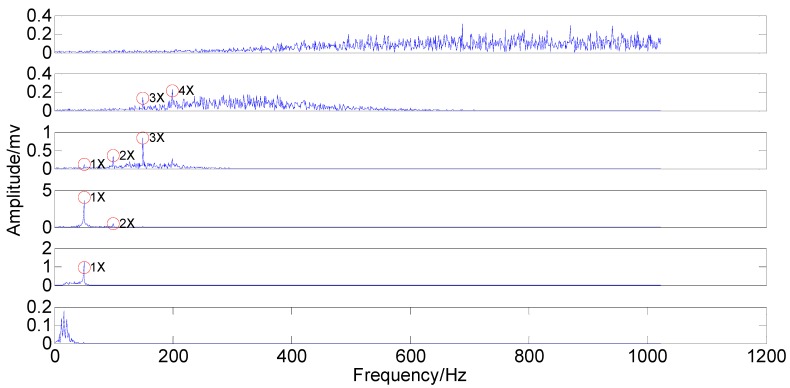
Optimized IMFs in the frequency-domain.

For comparison, the rubbing signal is also analyzed, both by the original EEMD and the optimized EEMD, where the original amplitude of added noise is equal to 1.09 and the optimized one is 1.26. These two kinds of decompositions are shown in Figure 15, Figure 16, Figure 17 and Figure 18.

Comparing the original IMFs in Figure 15 and Figure 16 with the optimized IMFs in Figure 17 and Figure 18 indicate that the two kinds of mode mixing are both serious like the signal simulation result. However, 3X and 4X of IMF2 (rich noise here) do not display a clear distinction, either by the original EEMD or by the optimized EEMD. Moreover, 1X (3.674) of IMF4 by the optimized method is smaller than that (4.024) of the original IMF4 while 1X (1.275) of IMF5 by the optimized method is larger than the original one (0.947). These two variations can be explained by the relationship between the fixed red circles and the moved blue spectral lines. Unlike the signal simulation result, the transfer phenomenon of the weak components caused by the adjustment of the parameter consistently exists. In addition, the optimized *ε* only enables some information transfer rather than eliminating mode mixing. Neither the original EEMD nor the optimized EEMD can accurately extract the independent information from the investigated signal.

**Figure 19:**
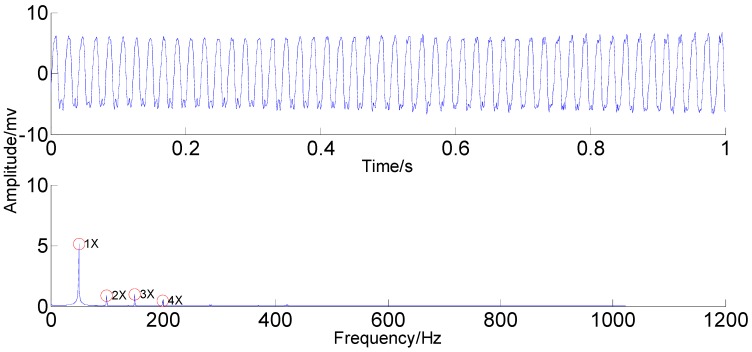
Reconstruction of rubbing signal.

ON the basis of the analyses above, it seems that PSEEMD is more accurate than the original EEMD and the optimized EEMD in extracting the independent and weak information. For final confirmation of PSEEMD, MIMFs 3–6 are applied for reconstructing the rubbing signal. The result depicted in Figure 19.

Compared with the original rubbing signal, the reconstructed signal retains the rubbing features of 1X, 2X, 3X and 4X; it eliminates the annoying noise and needless compositions due to the independent and weak information extraction capability of PSEEMD. The four components of 1X, 2X, 3X and 4X contained in the investigated signal are truly represented by the four single, intact and independent MIMFs. As a result, PSEEMD can still accurately extract the features independently and eliminate mode mixing although the complexity resulting from the non-linear and non-stationary features increases significantly compared with that of the simulation signal.

## 5. Conclusions

The independent extraction model of mixing features reconstructed in phase space named PSEEMD has been proposed to improve mode mixing of EEMD and realize the independent extraction of the frequency information. PSEEMD has been applied in vibration analysis of rotating machinery. Moreover, the theoretical analyses, the model construction, and the application have been performed. The following advantages of PSEEMD can be obtained.

1. Style mixing can be eliminated.
2. The change in the amplitude of added noise is robust.
3. The weak features can be extracted independently.
4. The advantages of (1)–(3) are realized under the condition of suffering from strong noise.
5. PSEEMD may have the generalization capability. It is expected to apply PSEEMD in analyzing non-linear and non-stationary signals in other fields.

Further study on the verification of PSEEMD application in rubbing coupled with other faults should be conducted.
